# Rab40C is a novel Varp-binding protein that promotes proteasomal degradation of Varp in melanocytes

**DOI:** 10.1242/bio.201411114

**Published:** 2015-02-06

**Authors:** Ayaka Yatsu, Hikaru Shimada, Norihiko Ohbayashi, Mitsunori Fukuda

**Affiliations:** Laboratory of Membrane Trafficking Mechanisms, Department of Developmental Biology and Neurosciences, Graduate School of Life Sciences, Tohoku University, Aobayama, Aoba-ku, Sendai, Miyagi 980-8578, Japan

**Keywords:** Varp, Rab40C, Melanogenic enzyme, Degradation, Membrane traffic

## Abstract

Varp (VPS9-ankyrin repeat protein) was originally identified as an activator of small GTPase Rab21 through its VPS9 domain, but it has subsequently been shown to function as a Rab32/38 effector through its first ANKR1 domain. Although these functions of Varp are important for melanogenesis, Varp contains a second ANKR2 domain, whose function remained completely unknown. Here we identified Rab40C, an atypical Rab containing a SOCS box that recruits a ubiquitin ligase complex, as a novel ANKR2-binding protein and investigated its involvement in melanogenic enzyme trafficking in melanocytes. The results showed that overexpression of Rab40C in melanocytes caused a dramatic reduction in melanogenic enzyme Tyrp1 signals by promoting proteasomal degradation of Varp in a SOCS-box-dependent manner and that knockdown of Rab40C in melanocytes caused an increase in the amount of Varp. Intriguingly, Rab40C knockdown also caused a dramatic reduction in Tyrp1 signals, the same as Varp overexpression did. These findings indicated that Rab40C is a previously unexpected regulator of Tyrp1 trafficking in melanocytes through controlling the proteasomal degradation of Varp.

## Introduction

Melanosomes are specialized organelles that synthesize and store melanin pigments in melanocytes (Marks and Seabra, 2001; Raposo and Marks, 2007). Since they share some features with endosomes and lysosomes, e.g., a low pH, they are classified as lysosome-related organelles, a group of cell type-specific or tissue-specific subcellular compartments (Marks et al., 2013). Proper biogenesis and transport of melanosomes within melanocytes are crucial to pigmentation of the hair and skin of mammals, because genetic defects in these processes have been reported to cause a group of hereditary diseases, called albinism, which is characterized by hypopigmentation of the hair and skin, e.g., Griscelli syndrome (GS), Hermansky-Pudlak syndrome (HPS), and Chédiak-Higashi syndrome (Tomita and Suzuki, 2004; Di Pietro and Dell'Angelica, 2005; Van Gele et al., 2009). Genetic analyses of patients with these syndromes and their corresponding murine models in the past decade have revealed a variety of causative genes, but the precise function of most of the products of these genes in melanogenesis has yet to be determined.

Cumulative recent evidence has indicated that several members of the Rab-type small GTPase family, a family of conserved membrane trafficking proteins in all eukaryotes (Fukuda, 2008; Stenmark, 2009; Pfeffer, 2013), and their regulators play pivotal roles in the control of melanosome biogenesis and melanosome transport along cytoskeletons in mammalian epidermal melanocytes (reviewed in Ohbayashi and Fukuda, 2012). Rab27A, whose deficiency causes type 2 GS, was the first Rab protein discovered to be associated with pigmentation (Ménasché et al., 2000; Wilson et al., 2000), and it has been shown to regulate actin-dependent melanosome transport through formation of a tripartite protein complex with Slac2-a/melanophilin and myosin-Va (reviewed in Fukuda, 2005). Rab1A and Rab36 are involved in movements on microtubules through their roles in anterograde melanosome transport and retrograde melanosome transport, respectively (Ishida et al., 2012; Matsui et al., 2012), and Rab11B and Rab17 are involved in the melanosome transfer step from melanocytes to keratinocytes (Beaumont et al., 2011; Tarafder et al., 2014). Two closely related Rabs, Rab32 and Rab38, cooperatively regulate trafficking of melanogenic enzymes, e.g., tyrosinase and tyrosinase-related protein 1 (Tyrp1), to melanosomes in the melanosome biogenesis step (Wasmeier et al., 2006; Tamura et al., 2009; Tamura et al., 2011; Bultema et al., 2012). Because of the importance of Rab38 and its activator BLOC-3 in melanogenic enzyme trafficking (Loftus et al., 2002; Gerondopoulos et al., 2012), defects in Rab38 cause the diluted coat color of *chocolate* mice, and defects in BLOC-3 cause the hypopigmentation seen in type 1 and type 4 HPS patients.

We previously identified Varp (VPS9-ankyrin repeat protein) as a Rab32/38-specfic effector protein that specifically recognizes an active form of Rab32/38 and is involved in Tyrp1 trafficking to melanosomes together with Rab32/38 (Wang et al., 2008; Tamura et al., 2009; Tamura et al., 2011). Varp consists of at least four domains, an N-terminal VPS9 domain, which possesses Rab21-GEF (guanine nucleotide exchange factor) activity (Zhang et al., 2006; Ohbayashi et al., 2012), an ankyrin repeat 1 (ANKR1) domain, which binds Rab32/38 (Tamura et al., 2009; Tamura et al., 2011), a VID (VAMP7-interaction domain) domain, which binds VAMP7 (Burgo et al., 2009; Tamura et al., 2011; Schäfer et al., 2012), and a C-terminal ANKR2 domain, whose function in melanocytes, in contrast to the other three domains, was unknown.

In this study, we investigated whether the ANKR2 domain was also involved in Tyrp1 trafficking in melanocytes. We identified Rab40C as a novel ANKR2-domain-binding protein in melanocytes and found that manipulation of Rab40C either by RNAi-mediated knockdown or by overexpression altered the amount of Varp protein in melanocytes. We also found that the effect of Rab40C on Varp expression is completely dependent on the presence of a SOCS (suppressor of cytokine signaling) box in Rab40C that is associated with a ubiquitin ligase complex (Nicholson and Hilton, 1998; Lee et al., 2007). Based on our findings, we discuss an unexpected role of the Varp-ANKR2–Rab40C interaction in the quality control of Varp.

## Results

### Expression of the ANKR2 domain of Varp in melanocytes caused a dramatic reduction in Tyrp1 signals

**Fig. 1.**
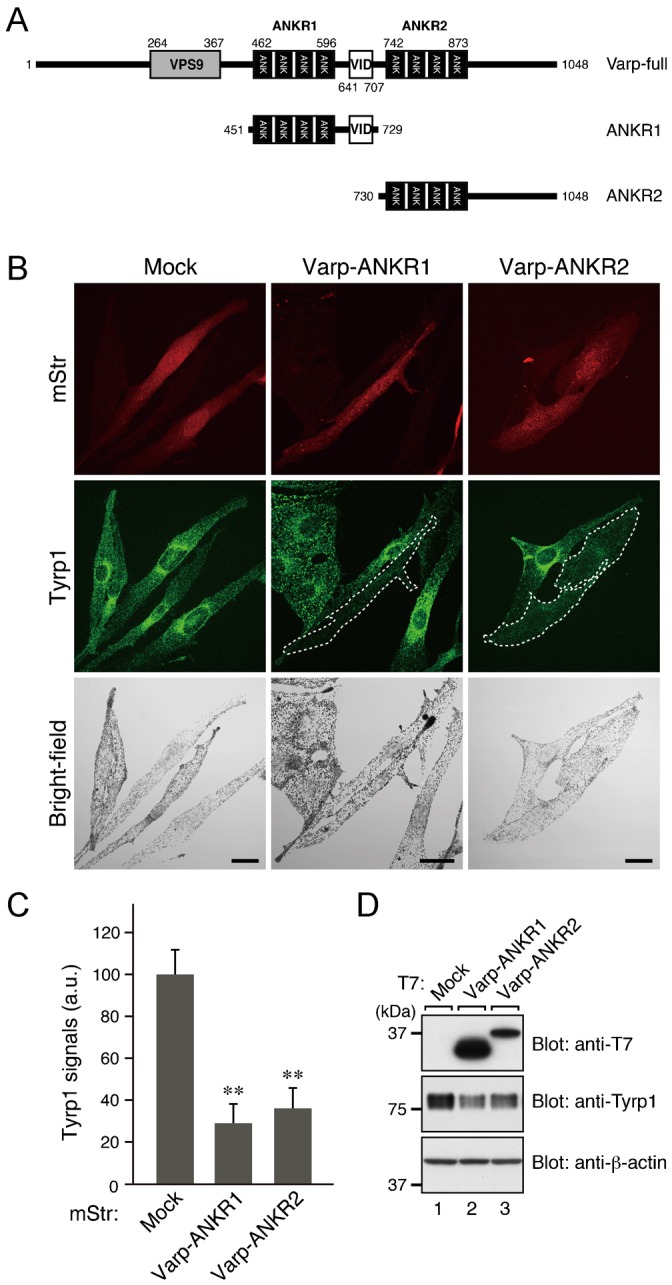
Effect of overexpression of the ANKR2 domain of Varp on Tyrp1 signals in melanocytes. (A) Schematic representation of the Varp mutants used in this study. Varp consists of an N-terminal VPS9 domain, which possesses Rab21-GEF activity (Zhang et al., 2006; Ohbayashi et al., 2012), and two C-terminal ankyrin repeat domains, named ANKR1 and ANKR2, respectively. A VID domain, which binds VAMP7, is present between the ANKR1 domain and ANKR2 domain (Burgo et al., 2009; Tamura et al., 2011; Schäfer et al., 2012). The ANKR1 domain and ANKR2 domain function as a Rab32/38-binding site (Tamura et al., 2009; Tamura et al., 2011) and a Rab40-binding site (this study), respectively. (B) Typical images of melanocytes expressing mStr-tagged Varp-ANKR1, Varp-ANKR2, and mStr alone (mStr fluorescence images, Tyrp1 images, and their corresponding bright-field images). Melan-a cells were transfected with pmStr-C1, pmStr-C1-Varp-ANKR1, or pmStr-C1-Varp-ANKR2 and then immunostained with anti-Tyrp1 mouse monoclonal antibody (middle row). Note that overexpression of either mStr-Varp-ANKR1 or mStr-Varp-ANKR2 in melan-a cells caused a dramatic reduction in Tyrp1 signals, suggesting that both domains are involved in Tyrp1 trafficking. mStr-Varp-ANKR1-expressing cells and mStr-Varp-ANKR2-expressing cells with reduced Tyrp1 signals are outlined with a broken line. Because the melanins that are already present are not immediately metabolized after the disappearance of melanogenic enzymes, there did not seem to be any difference in the melanin content of the ANKR1/2-expressing cells and control cells under our experimental conditions. Scale bars, 20 µm. (C) Quantification of the Tyrp1 signals shown in B. The bars represent the means and S.E. of data (28 cells for each mStr construct) from one representative experiment. **p<0.01, Dunnett's test. The results of three independent experiments are shown in supplementary material Fig. S5A. (D) Expression of T7-Varp-ANKR1 and T7-Varp-ANKR2 in B16-F1 cells as revealed by immunoblotting with the antibodies indicated. The positions of the molecular mass markers (in kDa) are shown on the left.

To investigate the possible involvement of the ANKR2 domain of Varp in the trafficking of melanogenic enzymes in melanocytes, we overexpressed the ANKR2 domain of Varp with mStr (monomeric Strawberry)-tag in melanocytes and observed its effect on the signals of the melanogenic enzyme Tyrp1. If the ANKR2 domain was actually involved in the control of Tyrp1 trafficking, the same as the ANKR1 domain is, its overexpression in melanocytes would reduce Tyrp1 signals, the same as ANKR1 overexpression did (Tamura et al., 2009) (Fig. 1B, middle panels). As anticipated, ANKR2 overexpression caused a dramatic reduction in Tyrp1 signals in melanocytes (Fig. 1B, right panels), the same as ANKR1 overexpression did (Fig. 1C). The reduced level of Tyrp1 protein expression in the ANKR2-overexpressing cells was confirmed by immunoblotting (Fig. 1D).

### Identification and characterization of Rab40C as a novel Varp-binding protein

**Fig. 2.**
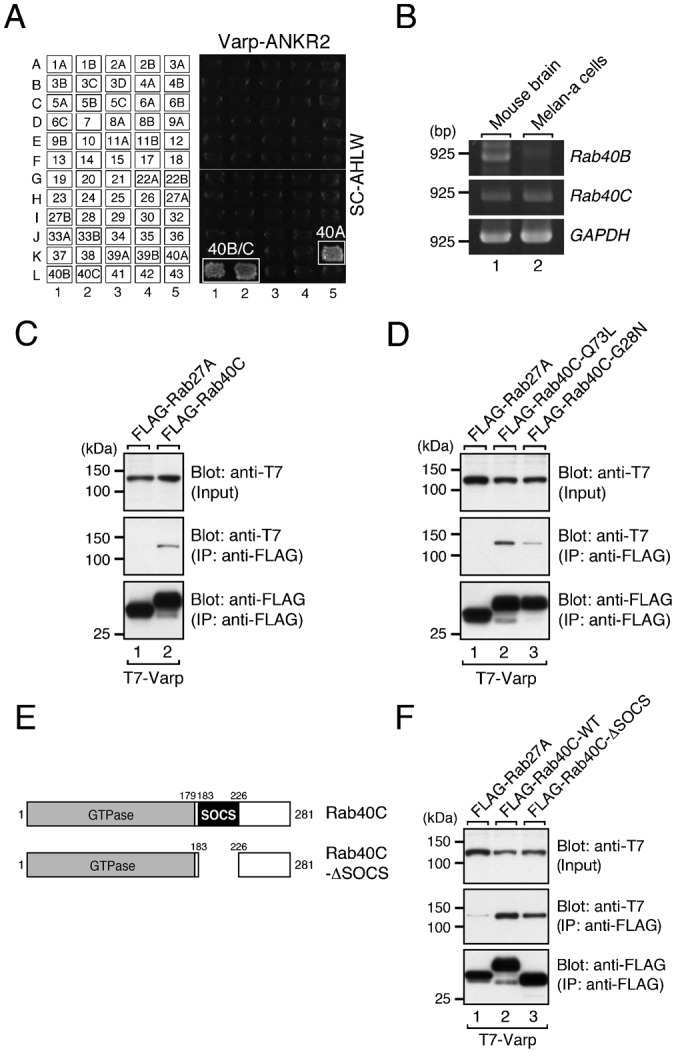
Identification and characterization of Rab40C as a novel Varp-binding protein. (A) Rab binding specificity of the ANKR2 domain of Varp as revealed by yeast two-hybrid panels. Yeast cells containing pGBD plasmid expressing each of 60 constitutive active Rabs (positions indicated in the left panels) (Fukuda et al., 2008) and pAct2 plasmid expressing the ANKR2 domain of Varp were streaked on SC-AHLW and incubated at 30°C for 1 week. Positive patches are boxed. Note the specific interactions between Varp-ANKR2 and Rab40A–C. (B) Expression of *Rab40C* mRNA, but not *Rab40B* mRNA, in melan-a cells as revealed by an RT-PCR analysis. *GAPDH* mRNA expression (bottom panel) is shown as a control to ensure that equivalent amounts of first-strand cDNA were used for the RT-PCR analysis. The size of the molecular mass markers (bp, base pair) is shown on the left side of the panel. (C) Full-length Varp interacts with Rab40C in COS-7 cells. (D) Varp preferably interacts with Rab40C-Q73L, a constitutive active form, over Rab40C-G28N, a constitutive negative form, in COS-7 cells. (E) Schematic representation of Rab40C and its mutant (Rab40C-ΔSOCS) used in this study. In contrast to classical Rab isoforms, members of the Rab40 subfamily contain a SOCS box, which interacts with Cullin5, a subunit of a ubiquitin ligase complex (Nicholson and Hilton, 1998; Lee et al., 2007), in addition to containing a GTPase domain. (F) Varp interacts with Rab40C-ΔSOCS in COS-7 cells. Co-immunoprecipitation assays in COS-7 cells were performed as described under “Materials and Methods”. Input means 1/100 volume of the reaction mixture used for immunoprecipitation (top panels). The positions of the molecular mass markers (in kDa) are shown on the left in C, D, and F.

Because ankyrin repeats often serve as a protein interaction site (Li et al., 2006), we hypothesized that the ANKR2 domain of Varp also binds a specific ligand(s) and that the ANKR2 domain alone functions as a dominant negative construct that disrupts the endogenous ANKR2–ligand interaction in melanocytes. To identify a candidate ligand for the ANKR2 domain, we initially focused on the small GTPase Rabs for the following two reasons. Our first reason was that several ankyrin repeat (ANKR) domains have recently been shown to function as a specific Rab-binding site, e.g., the ANKR domain of ORP1L interacts with Rab7 (Johansson et al., 2005), the ANKR domain of centaurin-β2/ACAP-2 with Rab35 (Kobayashi and Fukuda, 2012), and the ANKR1 domain of Varp with Rab32/38 (Tamura et al., 2009). Our second reason was that the ANKR1 domain (Rab32/38-binding site) and ANKR2 domain exhibit relatively high sequence similarity (61% amino acid similarity) (Tamura et al., 2009). To test our hypothesis, we investigated all of the possible interactions between the ANKR2 domain and 60 different mammalian Rabs by performing yeast two-hybrid assays (Fukuda et al., 2008). Intriguingly, the ANKR2 domain specifically interacted with three Rab40 isoforms, Rab40A–C (Fig. 2A; supplementary material Fig. S1). Since the results of the reverse transcription (RT)-PCR analysis indicated that Rab40C, but not Rab40B, was expressed in mouse melan-a cells (Fig. 2B) and Rab40A was not retained in mice (Itoh et al., 2006), we focused on the Rab40C isoform in our subsequent analysis in melanocytes.

We performed co-immunoprecipitation assays in COS-7 cells to determine whether the interaction between full-length Varp and Rab40C occurs in mammalian cells and, as shown in Fig. 2C, T7-tagged full-length Varp interacted with FLAG-tagged Rab40C. Moreover, T7-Varp preferably interacted with the constitutive active mutant Rab40C-Q73L over the constitutive negative mutant Rab40C-G28N (Fig. 2D, middle panel, lanes 2 and 3), indicating that Varp preferentially recognizes the GTP-bound active form of Rab40C. Interaction between FLAG-tagged Rab40C and endogenous Varp in melanocytes was also confirmed by co-immunoprecipitation assays (supplementary material Fig. S2).

We next focused our attention on the Varp-binding site in Rab40C, because members of the Rab40 subfamily are unique in containing a SOCS box, which is associated with a ubiquitin ligase complex (Nicholson and Hilton, 1998; Lee et al., 2007) (Fig. 2E). To investigate the involvement of the SOCS box of Rab40C in Varp binding, we produced a SOCS box deletion mutant, named Rab40C-ΔSOCS, and evaluated its Varp binding ability by co-immunoprecipitation assays as described above. However, the Rab40C-ΔSOCS mutant normally interacted with Varp, the same as the wild-type Rab40C did (Fig. 2F, lanes 2 and 3 in the middle panel). Taken together, these results indicated that the ANKR2 domain of Varp interacts with the GTPase domain of Rab40C rather than with its SOCS box.

### Effect of Rab40C overexpression on the level of Varp expression in melanocytes

**Fig. 3.**
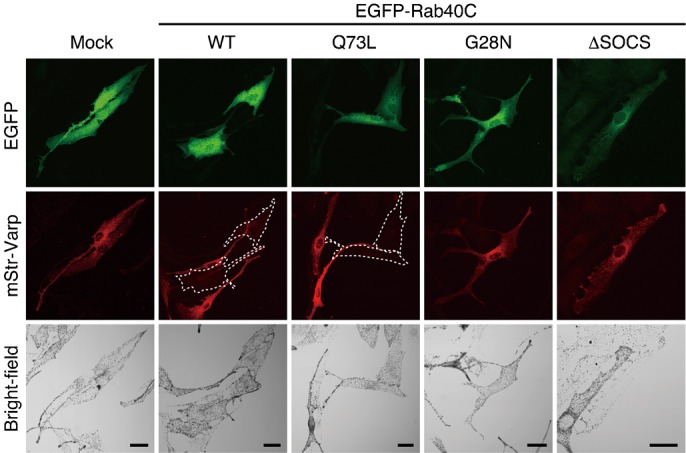
EGFP-Rab40C suppresses mStr-Varp expression in melanocytes. Typical images of melanocytes expressing EGFP-tagged Rab40C (WT, Q73L, G28N, or ΔSOCS) and mStr-tagged Varp (EGFP/mStr fluorescence images and their corresponding bright-field images). Melan-a cells were transfected with pEGFP-C1, pEGFP-C1-Rab40C-WT, pEGFP-C1-Rab40C-Q73L, pEGFP-C1-Rab40C-G28N, or pEGFP-C1-Rab40C-ΔSOCS together with pmStr-C1-Varp and then analyzed with a confocal fluorescence microscope. Note that mStr-Varp expression was often suppressed when coexpressed with EGFP-Rab40C-WT or EGFP-Rab40C-Q73L. EGFP-Rab40C-WT-expressing cells and EGFP-Rab40C-Q73L-expressing cells with reduced mStr-Varp signals are outlined with a broken line. Scale bars, 20 µm.

**Fig. 4.**
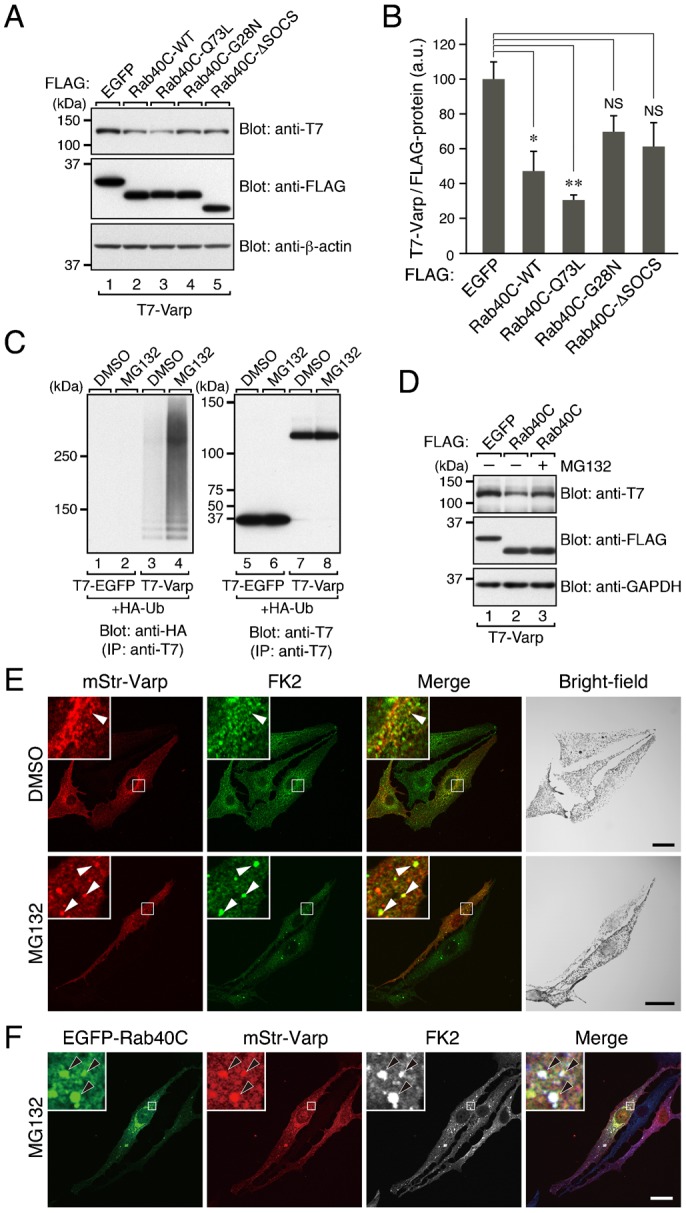
Rab40C promotes degradation and ubiquitination of Varp in melanocytes. (A) Expression of FLAG-tagged Rab40C-WT and Rab40C-Q73L, but not of Rab40C-G28N, Rab40C-ΔSOCS, or EGFP, decreased the amount of T7-Varp in B16-F1 cells. (B) Quantification of the T7-Varp bands shown in the top panel in A. The bars represent the means and S.E. of data from three independent experiments. *p<0.05; **p<0.01 in comparison with EGFP (Dunnett's test). NS, not significant. (C) Ubiquitination of Varp. T7-Varp (or T7-EGFP) was coexpressed with HA-tagged ubiquitin (Ub) in B16-F1 cells. After the cells were exposed to DMSO or MG132, T7-tagged proteins were immunoprecipitated with anti-T7 tag antibody-conjugated agarose beads followed by immunoblotting with the antibodies indicated. (D) Exposure of B16-F1 cells expressing T7-Varp together with FLAG-tagged Rab40C or EGFP to MG132 (or DMSO). Note that the decrease in the amount of T7-Varp in the Rab40C-coexpressing cells was reversed by MG132 exposure (compare lanes 2 and 3). The positions of the molecular mass markers (in kDa) are shown on the left in A, C, and D. (E) Varp was often colocalized with ubiquitin in melan-a cells exposed to the proteasome inhibitor MG132. Melan-a cells transiently expressing mStr-Varp were exposed to DMSO (upper panels) or MG132 (lower panels) for 3 hours and then immunostained with anti-multi ubiquitin mouse monoclonal antibody (FK2). The arrowheads in the insets indicate the colocalization points between ubiquitin (FK2) and Varp, which are evident in the presence of MG132 (lower panels). (F) Colocalization of Rab40C, Varp, and ubiquitin in MG132-exposed melan-a cells. The arrowheads in the insets indicate colocalization of the three proteins (FK2 signals are shown in blue in the merged image). The insets show magnified views of the boxed areas. Scale bars, 20 µm.

To identify the site of colocalization between Varp and Rab40C at the cellular level, mStr-tagged Varp and EGFP-tagged Rab40C were coexpressed in melanocytes. To our surprise, however, we were unable to detect a sufficient amount of mStr-Varp signals when the level of EGFP-Rab40C expression was relatively high (Fig. 3, second panel from the left in the middle row, and supplementary material Fig. S3B). By contrast, strong mStr-Varp signals were easily detected, when EGFP alone was overexpressed in melanocytes (Fig. 3, far left panel in the middle row, and supplementary material Fig. S3A). A similar tendency toward suppression of Varp expression was also observed in response to Rab40C-Q73L, a constitutive active Rab40C mutant, which preferentially bound Varp (Fig. 2D and Fig. 3, middle panel in the middle row, and supplementary material Fig. S3C), whereas Rab40C-G28N, a constitutive negative mutant, which hardly bound Varp at all (Fig. 2D), had no effect on Varp expression (Fig. 3, second panel from the right in the middle row, and supplementary material Fig. S3D). Intriguingly, when the SOCS box of Rab40C was deleted, the Rab40C-ΔSOCS mutant, which still retained Varp binding ability (Fig. 2F), was expressed together with Varp, the same as EGFP alone (Fig. 3, far right panel in the middle row, and supplementary material Fig. S3E). The SOCS-box-dependent and Varp-interaction-dependent suppression of Varp expression by Rab40C mutants was confirmed by immunoblotting (Fig. 4A,B). The suppression of Varp protein expression cannot have been attributable to the decreased expression of *Varp* mRNA, because there was no difference in the level of *Varp* mRNA expression when Rab40C was overexpressed (data not shown).

As noted above, the SOCS box of Rab40 has been shown to interact with Cullin5, a subunit of a ubiquitin ligase complex (Nicholson and Hilton, 1998; Lee et al., 2007), and has been suggested to be involved in the ubiquitination of target proteins. Thus, it is highly possible that Rab40C promotes ubiquitination and degradation of Varp in a SOCS-box-dependent manner. As anticipated, Varp itself was ubiquitinated in melanocytes as demonstrated by coexpression of T7-tagged Varp together with HA-tagged ubiquitin (Ub) (Fig. 4C). Ubiquitinated Varp is likely to be degraded by proteasomes, because exposure to the proteasome inhibitor MG132 restored the amount of Varp in Rab40C-expressing melanocytes to the control level (Fig. 4D, lanes 1 and 3). Furthermore, colocalization between mStr-tagged Varp and Ub (or EGFP-tagged Rab40C) was often observed in MG132-exposed melanocytes (Fig. 4E, lower panels, arrowheads, and Fig. 4F, arrowheads). These results taken together indicated that Rab40C promotes ubiquitination and degradation of Varp through interaction with the ANKR2 domain in a SOCS-box-dependent manner.

### Effect of Rab40C overexpression on the Tyrp1 trafficking in melanocytes

**Fig. 5.**
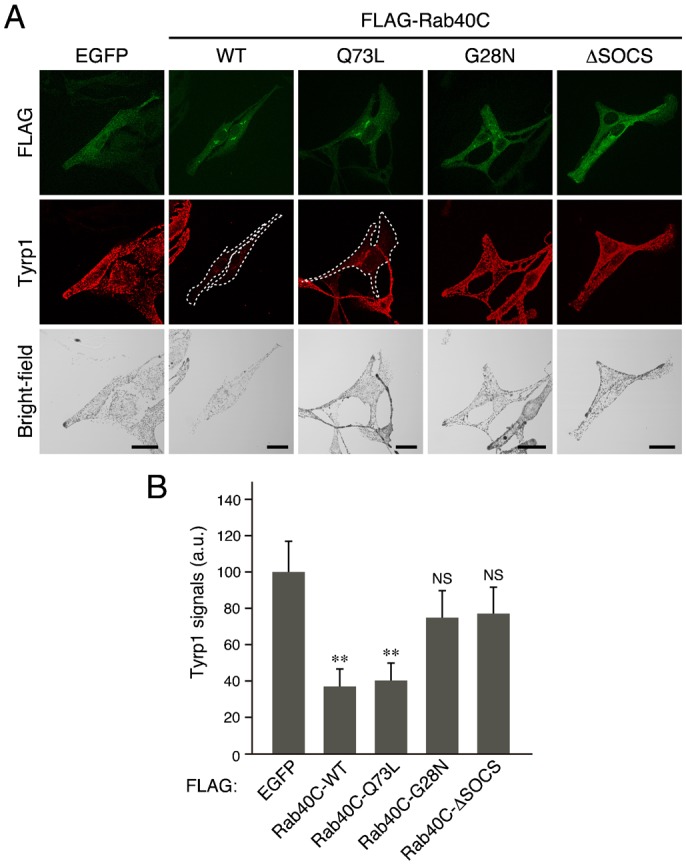
Effect of overexpression of Rab40C and its mutants on Tyrp1 signals in melanocytes. (A) Typical images of melanocytes expressing FLAG-tagged Rab40C (WT, Q73L, G28N, or ΔSOCS) (FLAG-EGFP/Rab40C images, Tyrp1 images, and their corresponding bright-field images). Melan-a cells were transfected with pEF-FLAG-EGFP or with pEF-FLAG-Rab40C (WT, Q73L, G28N, or ΔSOCS) and then immunostained with anti-FLAG tag mouse monoclonal antibody (top row) and anti-Tyrp1 rabbit polyclonal antibody (middle row). Note that overexpression of either FLAG-Rab40C-WT or FLAG-Rab40C-Q73L in melan-a cells caused a dramatic reduction in Tyrp1 signals. FLAG-Rab40C-WT-expressing cells and FLAG-Rab40C-Q73L-expressing cells with reduced Tyrp1 signals are outlined with a broken line. Scale bars, 20 µm. (B) Quantification of the Tyrp1 signals shown in A. The bars represent the means and S.E. of data (30 cells for each FLAG construct) from one representative experiment. **p<0.01, Dunnett's test. NS, not significant. The results of three independent experiments are shown in supplementary material Fig. S5B.

The reduced expression of Varp in Rab40C-overexpressing cells described above (Figs 3 and 4) prompted us to investigate whether Rab40C also affects trafficking of Tyrp1, one of the cargos of the Varp–Rab32/38 complex (Tamura et al., 2009; Tamura et al., 2011). Consistent with the reduced amount of Varp, both Rab40C-WT and Rab40C-Q73L significantly reduced the amount of Tyrp1, but Rab40C-G28N or Rab40C-ΔSOCS had almost no effect on Tyrp1 signals (Fig. 5A,B), a finding that was consistent with the fact that neither Rab40C-G28N nor Rab40C-ΔSOCS affects the Varp expression level (Fig. 4B). We therefore concluded that the Rab40C overexpression-dependent reduction in the amount of Varp protein in melanocytes is sufficient to inhibit Tyrp1 trafficking to melanosomes, the same as Varp knockdown was (Tamura et al., 2009).

### Effect of Rab40C knockdown on the Varp expression level and Tyrp1 trafficking in melanocytes

**Fig. 6.**
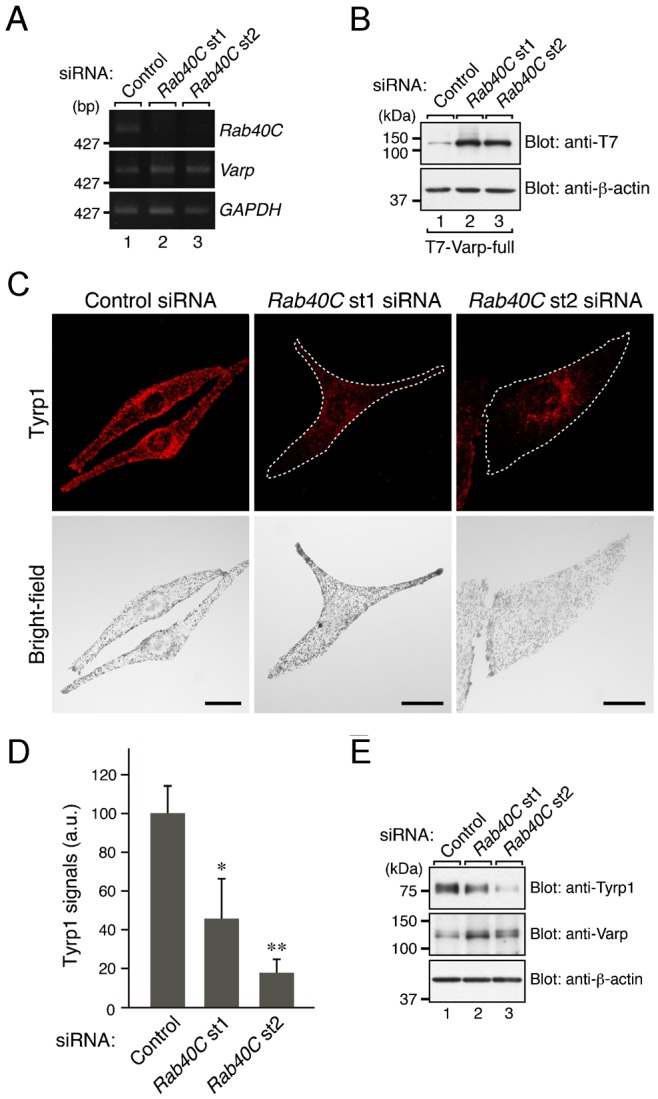
Effect of knockdown of Rab40C in melanocytes on the level of Varp expression and Tyrp1 expression. (A) Knockdown efficiency of *Rab40C* siRNAs as revealed by semi-quantitative RT-PCR analysis. Both *Rab40C* siRNAs (st1 and st2) dramatically suppressed the level of *Rab40C* mRNA expression (top panel) without affecting the level of *Varp* mRNA expression (middle panel). *GAPDH* mRNA expression (bottom panel) is shown as a control to ensure that equivalent amounts of first-strand cDNA were used for the RT-PCR analysis. The size of the molecular mass markers (bp, base pair) is shown on the left side of the panel. (B) Knockdown of Rab40C increased the amount of Varp protein in melanocytes. Cell lysates of B16-F1 cells that had been transfected with *Rab40C* siRNA (st1 or st2) and pEF-T7-Varp were subjected to SDS-PAGE followed by immunoblotting with the antibodies indicated. Note that the Rab40C-knockdown cells contained a greater amount of T7-Varp protein than the control cells (upper panel) even though their *Varp* mRNA level was unaltered (see A, middle panel). (C) Typical images of Rab40C-knockdown melanocytes (Tyrp1 images and their corresponding bright-field images). Melan-a cells were transfected with *Rab40C* siRNAs and then immunostained with anti-Tyrp1 mouse monoclonal antibody (upper panels). Note that Rab40C knockdown caused a dramatic reduction in Tyrp1 signals. Rab40C-knockdown cells are outlined with a broken line. Scale bars, 20 µm. (D) Quantification of the Tyrp1 signals shown in C. The bars represent the means and S.E. of data (30 cells for each siRNA) from one representative experiment. *p<0.05; **p<0.01, Dunnett's test. The results of three independent experiments are shown in supplementary material Fig. S5C. (E) Reduced expression of Tyrp1 and increased expression of Varp in Rab40C-knockdown melan-a cells as revealed by immunoblotting with the antibodies indicated. The positions of the molecular mass markers (in kDa) are shown on the left in B and E.

If Rab40C is a crucial factor in controlling the Varp expression level, Rab40C knockdown in melanocytes should also affect Varp expression. To determine whether it does, we knocked down endogenous Rab40C in melanocytes with specific siRNAs (small interfering RNAs) against *Rab40C* (Fig. 6A, top panel). Although Rab40C knockdown did not affect the expression of endogenous *Varp* mRNA in melanocytes (Fig. 6A, middle panel), it clearly resulted in an increase in the amount of both recombinant T7-Varp protein and endogenous Varp protein (Fig. 6B, upper panel and 6E, middle panel), in sharp contrast to the Rab40C overexpression, which resulted in a decrease in the amount of Varp protein (Fig. 4B). To our surprise, however, there was a dramatic reduction in Tyrp1 signals in the Rab40C-knockdown cells (Fig. 6C–E), the same as in the Rab40C-overexpressing cells (Fig. 5). These observations were unlikely to be attributable to off-target effects of siRNAs, because the Rab40C-knockdown effects described in Fig. 6B–E were observed in response to two independent *Rab40C* siRNAs.

**Fig. 7.**
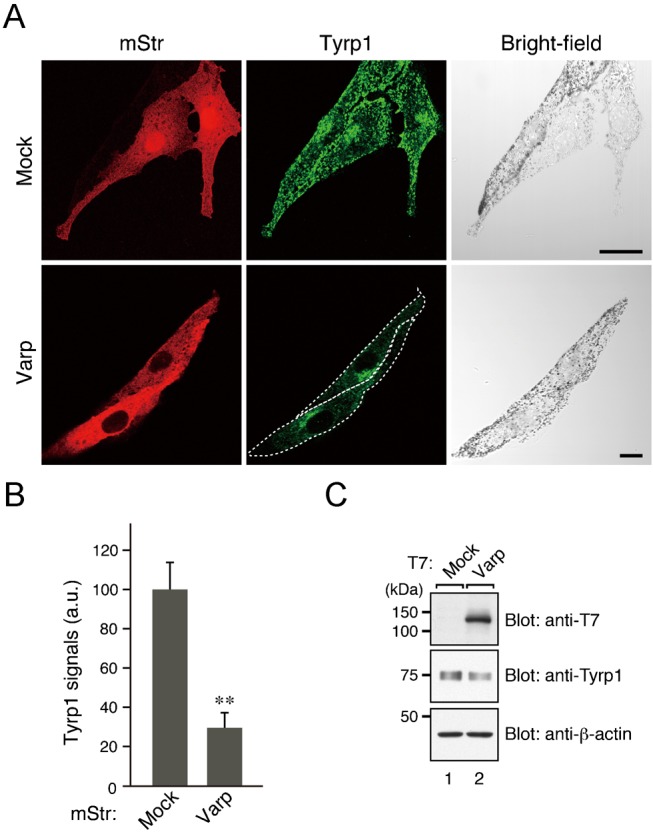
Effect of overexpression of Varp on Tyrp1 signals in melanocytes. (A) Typical images of mStr-Varp-expressing melanocytes (Tyrp1 images and their corresponding bright-field images). mStr-Varp-expressing melan-a cells were immunostained with anti-Tyrp1 mouse monoclonal antibody (middle panels). Note that Varp overexpression caused a dramatic reduction in Tyrp1 signals. Varp-overexpressing cells are outlined with a broken line. Scale bars, 20 µm. (B) Quantification of the Tyrp1 signals shown in A. The bars represent the means and S.E. of data (30 cells for each mStr construct) from one representative experiment. **p<0.01, Student's unpaired *t*-test. The results of three independent experiments are shown in supplementary material Fig. S5D. (C) Reduced expression of Tyrp1 in Varp-overexpressing B16-F1 cells as revealed by immunoblotting with the antibodies indicated. The positions of the molecular mass markers (in kDa) are shown on the left.

To determine whether the increased amount of Varp protein in Rab40C-knockdown cells was a major cause of the reduction in Tyrp1 signals, we finally investigated the effect of overexpression of T7-tagged Varp on Tyrp1 signals in control melanocytes. As anticipated, expression of full-length Varp in melanocytes also dramatically reduced Tyrp1 signals (Fig. 7), the same as expression of truncated ANKR2 (or ANKR1) domain of Varp did (Fig. 1). These results indicated that the reduction in Tyrp1 signals in Rab40C-knockdown cells was mainly attributable to the increase in Varp molecules (Fig. 7C).

## Discussion

We previously showed that Varp regulates not only Tyrp1 trafficking to melanosomes through interaction with Rab32/38 via its ANKR1 domain (Tamura et al., 2009; Tamura et al., 2011) but also regulates dendrite formation through activation of Rab21 via its VPS9 domain (Ohbayashi et al., 2012). However, involvement of the ANKR2 domain of Varp in melanogenesis had never been investigated. In the present study, we for the first time investigated involvement of the ANKR2 domain of Varp in Tyrp1 trafficking to melanosomes (Fig. 1) and succeeded in identifying Rab40A/B/C as a novel ANKR2-binding protein (Fig. 2). We showed by overexpression and knockdown experiments that Rab40C is actually required for proper localization of Tyrp1 at melanosomes (Figs 5 and 6). Although Varp preferentially interacts with the active form of Rab40C (Fig. 2D), in contrast to its established function as a Rab32/38 effector (Tamura et al., 2009; Tamura et al., 2011), Varp is unlikely to function as a Rab40C effector during Tyrp1 trafficking. Instead, Rab40C finely tunes the Varp expression level in melanocytes through regulation of the ubiquitination and degradation of Varp (Fig. 4A–D).

How does Rab40C promote the ubiquitination and degradation of Varp in melanocytes? Unlike the conventional Rabs, Rab40 isoforms have a SOCS box in their C-terminal region (Fig. 2E), and because of its presence they are ∼50 amino acids longer than conventional Rabs (Itoh et al., 2006; Lee et al., 2007). Proteins containing a SOCS box are known to function as the substrate-recognition subunits of ECS-type (ElonginBC-Cullin-SOCS-box) Cullin RING E3 ubiquitin ligase complexes, and it has previously been reported that *Xenopus* Rab40 binds the Cullin5 E3 ubiquitin ligase complex (Lee et al., 2007). Consistent with that previous report, deletion of the SOCS box from Rab40C completely canceled out the effect of Rab40C overexpression on the Varp protein level in melanocytes (Figs 3 and 4A,B) and, consequently, on their Tyrp1 protein level (Fig. 5). In addition, because overexpression of Rab40C-Q73L, but not of Rab40C-G28N, caused a significant reduction in the Varp protein level as well as the Tyrp1 protein level (Figs 3–4,5), Rab40C binding to Varp is required for the control of the Varp protein level. Thus, it is highly possible that rather than promoting conventional membrane traffic in melanocytes Rab40C mediates recognition of Varp by a Cullin E3 ubiquitin ligase complex. However, we cannot completely rule out the possibility that Rab40C (or other Rab40 isoforms) has an additional membrane trafficking function(s) in melanocytes, the same as in other cell types (Jacob et al., 2013; Tan et al., 2013).

Why accumulation of Varp molecules in Rab40C-knockdown melanocytes causes a reduction in Tyrp1 signals (Fig. 6) is an open question that should be answered in a future study. Because it has recently been reported that Varp interacts with a variety of molecules, including Rab32/38, VAMP7, retromer components VPS29 and VPS35 (Hesketh et al., 2014; McGough et al., 2014), GolginA4, and Kif5A (Burgo et al., 2012), it will be interesting to test the effect of these Varp ligands on Rab40C-dependent Varp degradation. We speculate that one possible physiological function of Rab40C is to reduce the excess amount of Varp protein via the ubiquitin–proteasome system to prevent accumulation of the Varp molecules, which leads to the reduction in Tyrp1 signals. Further extensive research will be necessary to resolve this matter.

In conclusion, we have demonstrated a novel function of the ANKR2 domain of Varp, i.e., Rab40C-mediated ubiquitination of Varp in melanocytes, and that it contributes to Tyrp1 trafficking to melanosomes. This finding, together with our previous findings in regard to Varp, indicate that Varp is a pleiotropic regulator of three different types of Rabs in three different processes through three distinct domains, a regulator of Rab21 in dendrite formation through its VPS9 domain, a regulator of Rab32/38 in Tyrp1 trafficking through its ANKR1 domain, and a regulator of Rab40C in the control of its own degradation in melanocytes through its ANKR2 domain (summarized in supplementary material Fig. S4). The findings in this study provide new insights into the regulatory mechanism of melanogenic enzyme trafficking to melanosomes in melanocytes.

## Materials and Methods

### Materials

Anti-Tyrp1 mouse monoclonal antibody (Ta99) and anti-β-actin mouse monoclonal antibody were obtained from Santa Cruz Biotechnology (Santa Cruz, CA) and Applied Biological Materials (Richmond, Canada), respectively. Anti-multi ubiquitin mouse monoclonal antibody (FK2) and HRP (horseradish peroxidase)-conjugated anti-GAPDH (glyceraldehyde-3-phosphate dehydrogenase) mouse monoclonal antibody were from MBL (Nagoya, Japan). Anti-FLAG tag (M2) mouse monoclonal antibody and HRP-conjugated anti-FLAG tag (M2) mouse monoclonal antibody were from Sigma-Aldrich (St. Louis, MO). HRP-conjugated anti-T7 tag mouse monoclonal antibody and HRP-conjugated anti-HA tag (3F10) rat monoclonal antibody were from Merck Millipore (Darmstadt, Germany) and Roche Applied Science (Penzberg, Germany), respectively. Alexa 488/594-conjugated anti-mouse and/or anti-rabbit IgG goat antibodies were from Invitrogen (Carlsbad, CA). Anti-Tyrp1 rabbit polyclonal antibody was prepared as described previously (Yatsu et al., 2013). Anti-Varp guinea pig polyclonal antibody was raised against a peptide corresponding to the C-terminal 20 amino acids (amino acid residues 1029–1048) of mouse Varp, and then affinity-purified with beads that had been immobilized with the C-terminal 23 amino acids (amino acid residues 1026–1048) of Varp fused with GST (glutathione *S*-transferase) essentially as described previously (Fukuda and Mikoshiba, 1999). The proteasome inhibitor MG132 was obtained from Peptide Institute (Osaka, Japan). *N*-Ethylmaleimide was from Wako Pure Chemical Industries (Osaka, Japan). All other reagents used in this study were analytical grade or the highest grade commercially available.

### Plasmid construction

The cDNAs of mouse Rab40C [wild-type (WT), G28N, Q73L] and Varp were prepared as described previously (Tamura et al., 2009; Ishida et al., 2012; Ohbayashi et al., 2012). The cDNA of mouse ubiquitin (Ub) was amplified from mouse brain and testis cDNA (Clontech-Takara Bio, Shiga, Japan) by PCR with the following pair of oligonucleotides with a *BamHI* linker (underlined) or a stop codon (bold): Met primer, 5′-GGATCCATGCAGATCTTCGTGAAGACCCT-3′ and stop primer, 5′-ACAGCT**TTA**TTTGACCTTCTTCTTGG-3′. The cDNA of Ub was subcloned into the pEF-HA vector, which was modified from pEF-BOS (Fukuda, 2002). The deletion mutants of Varp were prepared as described previously (Tamura et al., 2009; Tamura et al., 2011). Unless otherwise specified Varp means full-length Varp throughout this paper. A Rab40C-ΔSOCS mutant (deletion of amino acid residues 183–225 of mouse Rab40C) was prepared by inverse PCR using pGEM-T-Rab40C as a template and the following pair of oligonucleotides: forward primer, 5′-ATGGCCAACGGCATGACCGC-3′ and reverse primer, 5′-GAGCAAGATACGGGAGAGCT-3′. siRNAs against mouse *Rab40C* (target site 1: 5′-AACTGCATGGCCTTCTTTGAA-3′ and target site 2: 5′-CCATCAAAAGCCACCTCAAG-3′) were chemically synthesized by Nippon Gene (Toyama, Japan).

### Yeast two-hybrid assay

Yeast two-hybrid assays were performed by using pGBD-C1-Rab(CA) (constitutive active) lacking the C-terminal geranylgeranylation site (ΔCys) and pAct2-Varp-ANKR2 (see Fig. 1A), pAct2-Varp-ANKR2 only, or pAct2-Varp-C (see supplementary material Fig. S1A) as described previously (Fukuda et al., 2008; Tamura et al., 2009). The yeast strain (pJ69-4A), selection medium SC-AHLW (synthetic complete medium lacking adenine, histidine, leucine, and tryptophan), culture conditions, and transformation protocol used are described in James et al. (James et al., 1996).

### RT-PCR analysis

Marathon-Ready mouse brain cDNA was obtained from Clontech-Takara Bio. The total RNA of melan-a cells transfected with *Rab40C* siRNA, control siRNA, or nothing was prepared with TRI-reagent (Sigma-Aldrich), and reverse transcription was performed by using ReverTra Ace® (Toyobo, Osaka, Japan) according to the manufacturer's instructions. The pairs of oligonucleotides used for amplification were: for Rab40B, forward primer, 5′-GGATCCATGATGAGCTCCCTGGGCAG-3′ and reverse primer, 5′-CTAAGAAATTTTGCAGCTGT-3′; for Rab40C, forward primer 1, 5′-AGATCTATGGGCACCCAGGGCAGTCC-3′ and reverse primer 1, 5′-CTAGGAGATCTTGCAGTTGC-3′ (Fig. 2B), and forward primer 2, 5′-GTGTACGACATCACCAACCG-3′ and reverse primer 2, 5′-GAGAGCAGTTCTGAGGTGGG-3′ (Fig. 6A); for Varp, forward primer, 5′-CAGCACTGAGGTTCAGGACA-3′ and reverse primer, 5′-ACTGGCATAAGGGGTGACAG-3′; and for GAPDH, forward primer 1, 5′-ATGGTGAAGGTCGGAGTCAA-3′ and reverse primer 1, 5′-GCCATGTAGACCATGAGGTC-3′ (Fig. 2B), and forward primer 2, 5′-ACCACAGTCCATGCCATCAC-3′ and reverse primer 2, 5′-TCCACCACCCTGTTGCTGTA-3′ (Fig. 6A). The cDNAs of Rab40B, Rab40C, Varp, and GAPDH were amplified by PCR performed with LA-Taq DNA polymerase, Ex-Taq DNA polymerase (Clontech-Takara Bio), KOD Plus DNA polymerase, and/or rTaq DNA polymerase (Toyobo, Osaka, Japan) under non-saturated conditions.

### Immunofluorescence analysis

The black-mouse-derived immortal melanocyte cell line melan-a (generous gift of Dorothy C. Bennett) was cultured as described previously (Bennett et al., 1987; Kuroda et al., 2003). Plasmids were transfected into melan-a cells by using FuGENE6 (Promega, Madison, WI) or Lipofectamine 2000 (Invitrogen) each according to its manufacturer's instructions. Two days after transfection, cells were fixed with 4% paraformaldehyde or 10% (w/v) trichloroacetic acid, permeabilized with 0.3% Triton X-100, stained with specific primary antibodies, and then visualized with Alexa-Fluor 488/594-conjugated secondary antibodies. The cells were examined for immunostaining signals with a confocal laser-scanning fluorescence microscope (Fluoview FV1000-D; Olympus, Tokyo, Japan). Under our experimental conditions, the transfection efficiency of pEGFP-C1-Rab40C (or mStr-C1-Varp) in melan-a cells was approximately 10%, and only the EGFP (or mStr)-positive cells were examined. Proteasomes were inhibited by exposing cells to the proteasome inhibitor MG132 for 3 hours before fixation. The images were processed with Adobe Photoshop software (CS5). Tyrp1 signals were quantitatively measured by capturing images of the transfected cells at random (28 cells and 30 cells were examined in Fig. 1C and other figures, respectively, for each construct) with the confocal microscope and quantifying the fluorescent signals of Tyrp1 with MetaMorph software (Molecular Devices, Sunnyvale, CA). Each experiment was performed independently at least three times (n = 3) and statistically analyzed (supplementary material Fig. S5). Data representative of the results of the independent experiments are also shown in Fig. 1C, Fig. 5B, Fig. 6D, and Fig. 7B. The statistical analyses were performed by using Student's unpaired *t*-test or Dunnett's test (for multiple comparisons), and *p*-values <0.05 were considered statistically significant (*p<0.05; **p<0.01).

### Immunoblotting

B16-F1 cells were co-transfected with pEF-T7-Varp together with control siRNA or *Rab40C* siRNA by using Lipofectamine 2000, and melan-a cells were transfected with control siRNA or *Rab40C* siRNA by using Lipofectamine RNAiMAX (Invitrogen) according to the manufacturer's instructions. At 48 hours after transfection the B16-F1 cells (or melan-a cells) were washed with ice-cold PBS, scraped, and recovered by centrifugation at 800 ***g*** for 5 minutes at 4°C. The cells were lysed with the lysis buffer [50 mM HEPES-KOH pH 7.2, 150 mM NaCl, 1 mM MgCl_2_, and 1% Triton X-100 supplemented with complete EDTA-free protease inhibitor cocktail (Roche Applied Science)], and the lysates were subjected to 10% SDS-PAGE (or 5% SDS-PAGE for Fig. 4C) and transferred to a PVDF membrane. The blots were blocked with 1% skim milk or 1% BSA in PBS containing 0.1% Tween-20 and incubated with primary antibodies for 1 hour at room temperature and then with appropriate HRP-conjugated secondary antibodies for 1 hour at room temperature. Immunoreactive bands were detected by enhanced chemiluminescence (ECL, GE Healthcare, Little Chalfont, UK). The intensity of the immunoreactive bands was measured with ImageJ software. Proteasomes were inhibited by exposing cells to the proteasome inhibitor MG132 (10 nM) for 20 hours before harvesting them. The cells were lysed with 50 mM HEPES-KOH pH 7.2, 150 mM NaCl, 1 mM MgCl_2_, 0.1% (w/v) sodium deoxycholate, 1% (w/v) SDS, and 10 mM *N*-ethylmaleimide supplemented with complete EDTA-free protease inhibitor cocktail by passing them through a 27-gauge needle. Inhibition of T7-Varp degradation was assessed by 10% SDS-PAGE followed by immunoblotting with HRP-conjugated anti-T7 tag antibody.

### Co-immunoprecipitation assay

COS-7 cells (2×10^5^ cells/35-mm dish) were co-transfected with pEF-T7-Varp and pEF-FLAG-Rab40C (WT, Q73L, G28N, or ΔSOCS) by using Lipofectamine LTX Plus (Invitrogen) according to the manufacturer's instructions. At 36 hours after transfection the cells were lysed with the lysis buffer (50 mM HEPES-KOH pH 7.2, 150 mM NaCl, 1 mM MgCl_2_, and 1% Triton X-100 supplemented with complete EDTA-free protease inhibitor cocktail). Co-immunoprecipitation assays with anti-FLAG tag antibody-conjugated agarose beads (Sigma-Aldrich) were performed as described previously (Fukuda et al., 1999; Fukuda and Kanno, 2005). Ubiquitination of Varp was assessed by co-transfecting pEF-T7-Varp and pEF-HA-ubiquitin (Ub) into B16-F1 cells (5×10^5^ cells/60-mm dish) by using Lipofectamine LTX Plus according to the manufacturer's instructions. Proteasomes were inhibited by exposing the cells to the proteasome inhibitor MG132 (10 µM) for 3 hours before harvesting them. After lysing the cells with the lysis buffer containing 10 mM *N*-ethylmaleimide, T7-Varp was immunoprecipitated with anti-T7 tag antibody-conjugated agarose beads (Merck Millipore). Proteins bound to the beads were analyzed by 5% SDS-PAGE followed by immunoblotting with HRP-conjugated anti-T7 tag antibody and HRP-conjugated anti-HA tag antibody.

## Supplementary Material

Supplementary Material
