# Supplementary material for: Rab40C is a novel Varp-binding protein that promotes proteasomal degradation of Varp in melanocytes
Source: Biol Open. 2015 Feb 6;4(3):267–75. doi: 10.1242/bio.201411114 (PMC4359733; doi:10.1242/bio.201411114)
Supplement: Supplementary Material [file supp_4_3_267__index.html]

Rab40C is a novel Varp-binding protein that promotes proteasomal degradation of Varp in melanocytes — Rab40C is a novel Varp-binding protein that promotes proteasomal degradation of Varp in melanocytes — Supplementary Material 

# Rab40C is a novel Varp-binding protein that promotes proteasomal degradation of Varp in melanocytes

## bio.201411114 Supplementary Material

**Files in this Data Supplement:**

- Supplementary Material - Ayaka Yatsu et al. doi: 10.1242/bio.201411114
