# Supplementary material for: Rab40C is a novel Varp-binding protein that promotes proteasomal degradation of Varp in melanocytes
Source: Biol Open. 2015 Feb 6;4(3):267–75. doi: 10.1242/bio.201411114 (PMC4359733; doi:10.1242/bio.201411114)
Supplement: Supplementary Material [file supp_bio.201411114_bio.201411114-s1.pdf]

Biology Open (2015) 000, 1–9 doi:10.1242/bio.201411114

Supplementary Material
Ayaka Yatsu et al. doi: 10.1242/bio.201411114

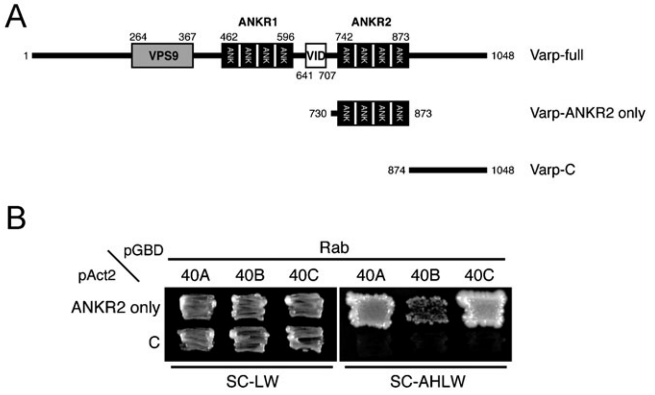

Fig. S1. The ANKR2 domain of Varp functions as a Rab40-binding site. (A) Schematic representation of the Varp-ANKR2 only and Varp-C constructs and (B) their Rab40 binding activity. Yeast cells containing pGBD plasmid expressing constitutive active Rab40A/B/C and pAct2 plasmid expressing the ANKR2 domain alone (Varp-ANKR2 only; amino acids 730–873) or the C-terminus (Varp-C; amino acids 874–1048) of Varp were streaked on SC-LW (left panels in B) and SC-AHLW (right panels in B) and incubated at 30˚C for one day and ten days, respectively. Note that Varp-ANKR2 construct alone is necessary and sufficient for Rab40-binding.

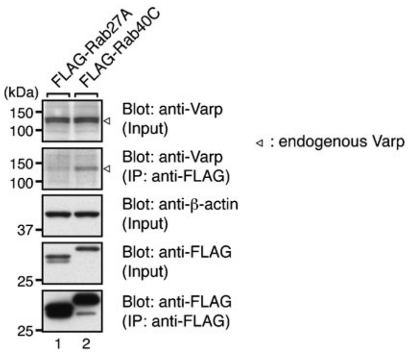

Fig. S2. Rab40C interacts with Varp in melanocytes. B16-F1 cells were transfected with pEF-FLAG-Rab40C (or pEF-FLAG-Rab27A as a negative control) by using Lipofectamine LTX Plus. At 16 hours after transfection the cells were exposed to 100 nM MG132 for 20 hours and then lysed with the lysis buffer. Co-immunoprecipitation assays were performed with anti-FLAG tag antibody-conjugated agarose beads. Proteins bound to the beads were analyzed by 10% SDS-PAGE followed by immunoblotting with the antibodies indicated. Input means 1/100 volume of the reaction mixture used for immunoprecipitation. The positions of the molecular mass markers (in kDa) are shown on the left.

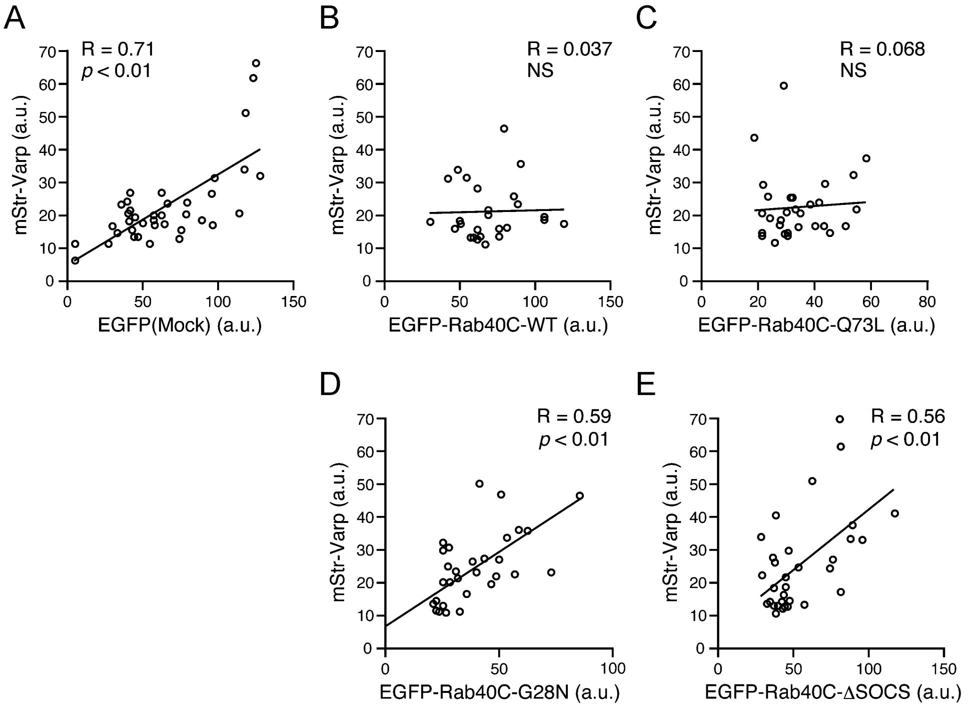

Fig. S3. Correlations between Rab40C expression and Varp expression in melanocytes. Scatter plots demonstrate whether the levels of mStr-Varp expression correlated with the levels of EGFP expression (A), EGFP-Rab40C-WT expression (B), EGFP-Rab40C-Q73L expression (C), EGFP-Rab40C-G28N expression (D), or EGFP-Rab40C-ΔSOCS expression (E) in melan-a cells. Immunofluorescence signals (more than 30 cells) were quantified with ImageJ software (version 1,47v; NIH, Bethesda, MD). Note that EGFP expression, Rab40-G28N expression, and Rab40C-ΔSOCS expression were significantly positively correlated with Varp expression, but that Rab40C-WT expression and Rab40C-Q73L expression were not correlated with Varp expression, because Rab40C-WT and Rab40C-Q73L expression promote degradation of Varp. R, Pearson's correlation coefficient; NS, not significant.

Biology Open (2015) 000, 1–9 doi:10.1242/bio.201411114

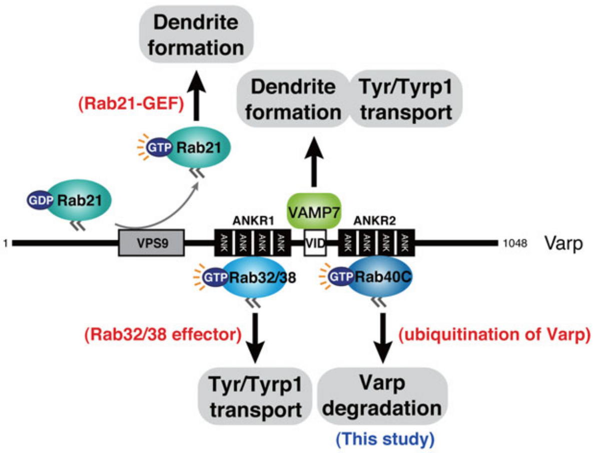

Fig. S4. Proposed functions of each domain of Varp in melanocytes. Varp is involved in dendrite formation through activation of Rab21 via the VPS9 domain (Ohbayashi et al., 2012). Varp also functions as a Rab32/38 effector that promotes transport of melanogenic enzymes, tyrosinase (Tyr) and Tyrp1, to melanosomes (Tamura et al., 2009; Tamura et al., 2011). The Varp–VAMP7 interaction is required for both dendrite formation and melanogenic enzyme transport (Tamura et al., 2011; Ohbayashi et al., 2012; Yatsu et al., 2013). Varp also interacts with Rab40C, which promotes ubiquitination and degradation of Varp (this study).

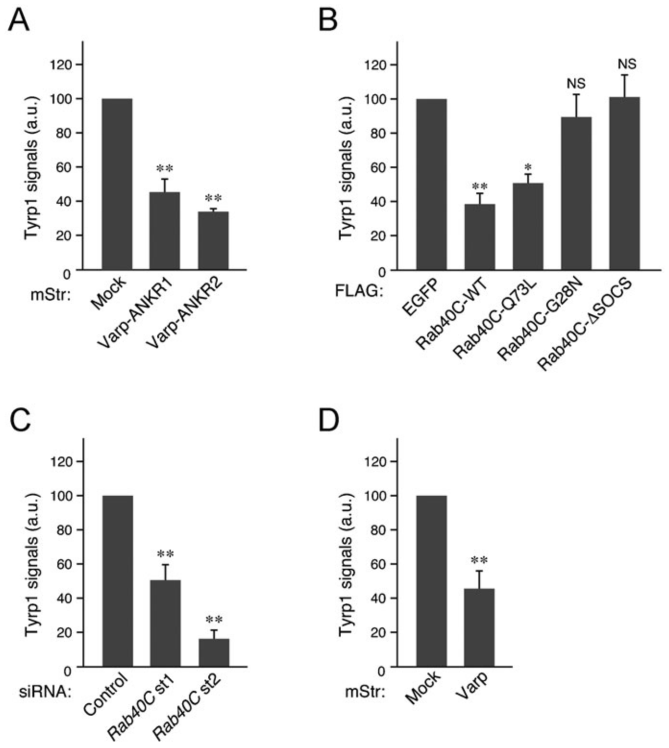

Fig. S5. Quantification of the Tyrp1 signals in melan-a cells shown in Fig. 1C (A), Fig. 5B (B), Fig. 6D (C), and Fig. 7B (D). The bars represent the means and S.E. of data from three independent experiments. *$p<0.05$; **$p<0.01$, Dunnett's test or Student's unpaired *t*-test. NS, not significant.
